# Application of an Accessible Interface for Pharmacokinetic Modeling and *In Vitro* to *In Vivo* Extrapolation

**DOI:** 10.3389/fphar.2022.864742

**Published:** 2022-04-13

**Authors:** David E. Hines, Shannon Bell, Xiaoqing Chang, Kamel Mansouri, David Allen, Nicole Kleinstreuer

**Affiliations:** ^1^ Inotiv-RTP, Research Triangle Park, Durham, NC, United States; ^2^ NIH/NIEHS/DNTP/NICEATM, Research Triangle Park, Durham, NC, United States

**Keywords:** physiologically–based pharmacokinetic model, *in vitro* to *in vivo* extrapolation, risk assessment, computational modeling methods, hazard screening, new approach methodology

## Abstract

Regulatory toxicology testing has traditionally relied on *in vivo* methods to inform decision-making. However, scientific, practical, and ethical considerations have led to an increased interest in the use of *in vitro* and in silico methods to fill data gaps. While *in vitro* experiments have the advantage of rapid application across large chemical sets, interpretation of data coming from these non-animal methods can be challenging due to the mechanistic nature of many assays. *In vitro* to *in vivo* extrapolation (IVIVE) has emerged as a computational tool to help facilitate this task. Specifically, IVIVE uses physiologically based pharmacokinetic (PBPK) models to estimate tissue-level chemical concentrations based on various dosing parameters. This approach is used to estimate the administered dose needed to achieve *in vitro* bioactivity concentrations within the body. IVIVE results can be useful to inform on metrics such as margin of exposure or to prioritize potential chemicals of concern, but the PBPK models used in this approach have extensive data requirements. Thus, access to input parameters, as well as the technical requirements of applying and interpreting models, has limited the use of IVIVE as a routine part of *in vitro* testing. As interest in using non-animal methods for regulatory and research contexts continues to grow, our perspective is that access to computational support tools for PBPK modeling and IVIVE will be essential for facilitating broader application and acceptance of these techniques, as well as for encouraging the most scientifically sound interpretation of *in vitro* results. We highlight recent developments in two open-access computational support tools for PBPK modeling and IVIVE accessible *via* the Integrated Chemical Environment (https://ice.ntp.niehs.nih.gov/), demonstrate the types of insights these tools can provide, and discuss how these analyses may inform in vitro-based decision making.

## Introduction

Historically, toxicity testing has relied on laboratory animal-based methods to inform decision-making. However, practical and ethical considerations and concern over the human biological relevance of some effects observed in animals has fueled interest in non-animal approaches (EPA, 2019). A 2016 amendment to the Toxic Substances Control Act by the United States Environmental Protection Agency (EPA) calls for the development of new approach methodologies (NAMs), such as *in vitro* assays and computational models, to meet the challenge of informing hazard and risk assessment while reducing dependence on animal testing (ICCVAM, 2018). In response, development of NAM applications for regulatory decision-making has been a priority for risk assessors (Craig et al., 2019; Parish et al., 2020) and regulatory agencies such as the US EPA (EPA, 2019).

**FIGURE 1:**
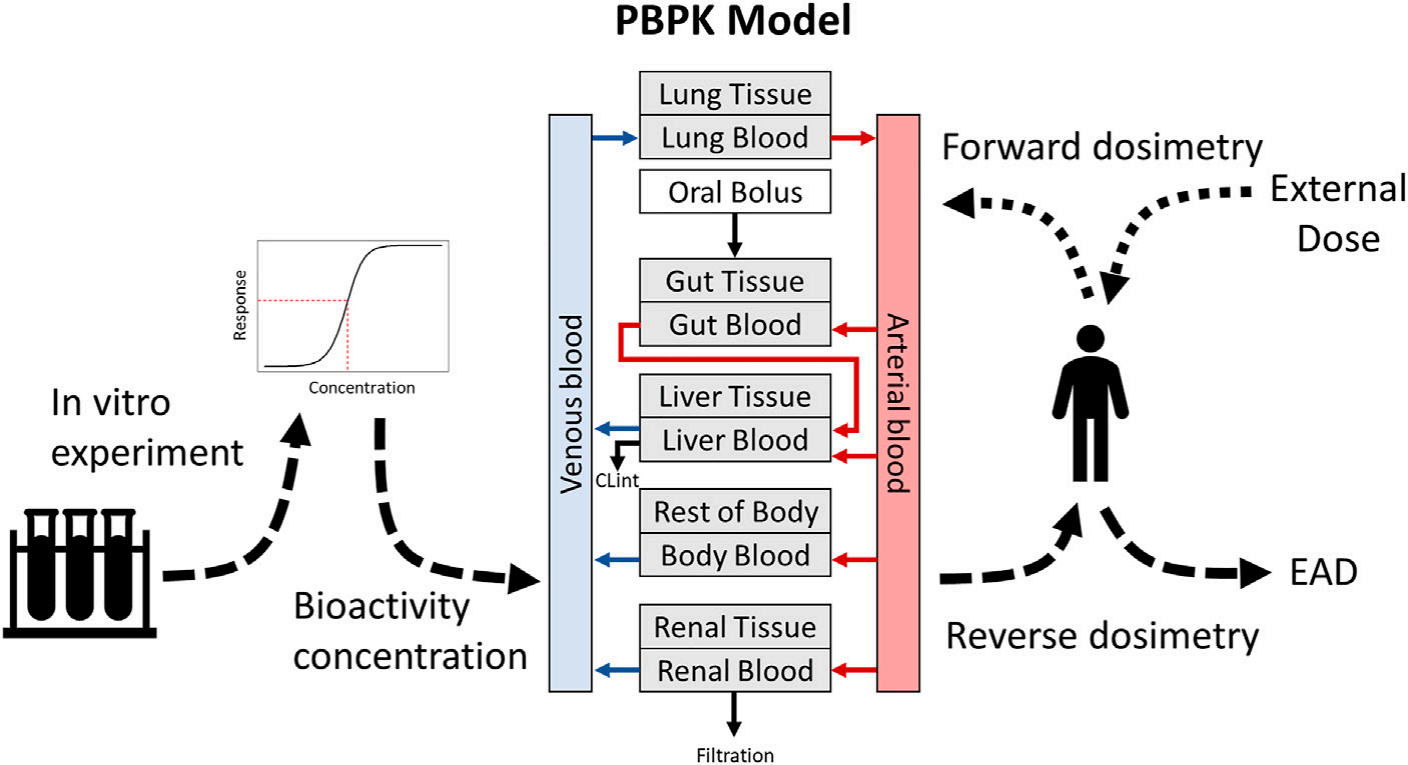
Overview of the uses of PBPK modeling for dose prediction and IVIVE. Dotted lines show forward dosimetry for predicting relevant doses from an external exposure, while dashed lines show reverse dosimetry for IVIVE and prediction of EADs.

*In vitro* assays have the potential to rapidly evaluate bioactivity across broad chemical sets. However, application of data from these assays to risk assessment requires translation of the bioactivity concentrations to exposures relevant at the organism level. The absorption, distribution, metabolism, and excretion (ADME) of chemicals within *in vivo* systems can influence toxicity. Therefore, results of *in vitro* tests need to be related back to the biological context where metabolism and redistribution occurs, which can be accomplished in part using *in vitro* to *in vivo* extrapolation (IVIVE). IVIVE makes use of physiologically based pharmacokinetic (PBPK) models to estimate relevant tissue concentrations (Cohen Hubal et al., 2019). These models apply reverse dosimetry to predict the equivalent administered dose (EAD) of a chemical that will result in tissue concentrations that match *in vitro* bioactivity concentrations (Yoon et al., 2012; Moxon et al., 2020). Additionally, PBPK models can be useful for informing the relevant concentrations and doses for *in vitro* and *in vivo* experiments by predicting target tissue concentrations from expected exposures. Thus, PBPK modeling and IVIVE are essential tools for experimental design and interpretation (Figure 1).

While PBPK models and IVIVE analyses can facilitate the interpretation of *in vitro* results, the application of these techniques requires chemical-specific ADME parameters, PBPK model equations, relevant *in vitro* data for endpoints of interest, and the technical knowledge to integrate this information. Data availability can limit applications, but increased access to predictions from quantitative structure-activity relationship (QSAR) models is helping to address data gaps. Additionally, open-access computational support tools for PBPK modeling and IVIVE promote the transparency and accessibility of these approaches. As regulatory application of NAMs become more common, tools that broaden the accessibility of IVIVE will be crucial for the use and interpretation of *in vitro* data.

Here we discuss approaches, data needs, and applications of PBPK modeling and IVIVE, as well as examples of open-access computational support tools that can help to democratize these analyses. We demonstrate how these techniques can place *in vitro* results in an *in vivo* context and why this context is important for interpretation when informing decision making. Further, we highlight potential regulatory applications and future directions for using *in vitro* and in silico NAMs in decision making.

## Physiologically Based Pharmacokinetic Modeling

Open-access PBPK modeling tools provide transparent use of generalized models designed to predict pharmacokinetics (PK) for a broad range of chemicals. This represents an advantage over commercial models, which are often tailored for pharmaceutical applications (Heimbach et al., 2009; Jamei et al., 2009).The EPA’s high throughput toxicokinetic (httk) R package, for example, is an open-access tool that can generate PK predictions for thousands of chemicals in generalized models of mice, rats, dogs, rabbits, monkeys, and humans (Pearce et al., 2017).

PBPK modeling packages such as httk often require technical knowledge and coding ability to operate. While powerful for customization of analyses, these requirements can create a barrier to exploratory PBPK applications and decrease accessibility for users who could benefit from rapid PBPK predictions. The National Toxicology Program’s Integrated Chemical Environment (ICE, https://ice.ntp.niehs.nih.gov/) is an open-access web tool that provides PBPK and IVIVE workflows *via* an intuitive graphical user interface. These tools provide a user-friendly front end and combine the httk package with parameter data to aid users in gaining comfort with modeling by reducing the technical knowledge requirements for conducting and interpreting analyses (Bell et al., 2020; Abedini et al., 2021).

All PBPK models require chemical-specific ADME and physicochemical parameters. For example, metabolism parameters such as intrinsic clearance (Obach, 2011; OECD, 2018), plasma protein fraction unbound (Poulin, Burczynski and Haddad, 2016; Hartung, 2018), and tissue-specific partition coefficients (Schmitt, 2008) are necessary and chemical structure-dependent. While availability of experimental data may be limited, QSAR modeling can help address this issue by providing parameter predictions where measured values are unavailable (Peyret and Krishnan, 2011; Zhang et al., 2018) and has the potential to broaden the applicability of PBPK approaches to all chemicals with defined structures (Escher et al., 2019).

ICE utilizes predictions generated by the Open (quantitative) structure–activity Relationship App (OPERA), a free and open-source/open-data suite of QSAR models developed and maintained under an ongoing collaboration between NICEATM and the EPA (Mansouri et al., 2018). OPERA provides “*in silico*” predictions for numerous physicochemical and ADME properties. OPERA also provides applicability domain assessment and accuracy estimates following established principles for QSAR validation (Tichý and Rucki, 2009; Mansouri et al., 2018, 2019). OPERA-generated ADME related properties used for PBPK modeling and IVIVE include intrinsic clearance, plasma protein fraction unbound, octanol-water partition coefficient, dissociation coefficient, Henry’s Law constant and the logarithmic acid dissociation constant. Details regarding the calculation and use of each of these parameter predictions can be found in the Supplementary Material S1. OPERA can be downloaded as either a command-line or graphical interface from the official NIEHS GitHub repository (https://github.com/NIEHS/OPERA).

To maximize approachability, ICE includes detailed walkthrough documentation and modifiable code in the form of R Notebooks (Abedini et al., 2021). The ICE PBPK tool allows users to select httk model type, species, exposure route, and dosing schedule to use exposure dose to predict *in vivo* internal concentrations (forward dosimetry). Furthermore, ICE allows for specification of physiochemical parameter sources (either measured, in silico, or default) to provide the users with control over the types of data being used for modeling. The default option is to use experimentally measured parameter values, where available, and predicted (in silico) values otherwise. Results provide PK time-series plots for up to eight different organs and tissues, as well as downloadable tables of model parameters, sources, and outputs. Tools such as ICE greatly increase the accessibility of PK prediction generation for chemical sets, and thus promote application of PBPK models in a broader range of studies, including those with regulatory applications (Sipes et al., 2017; Smith et al., 2020).

## *In Vitro* to *In Vivo* Extrapolation

IVIVE uses PBPK models to estimate tissue-level concentrations, then applies reverse dosimetry (*in vivo* internal concentration back to dose) to calculate EADs that correlate to *in vitro* bioactivity concentrations. Importantly, IVIVE accounts for some ADME processes that are not present *in vitro* to support interpretation of *in vitro* results in an *in vivo* context.

Applying IVIVE to high-throughput screening (HTS) data sets from programs such as Tox21 (Tice et al., 2013) and ToxCast (Kavlock et al., 2012) can enable rapid chemical risk screening and prioritization for large, diverse chemical sets. The availability of QSAR-parameterized PBPK models has made such applications practical (Breen et al., 2021). For example, the EPA’s Endocrine Disruptor Screening Program used an IVIVE approach combined with HTS data to develop an integrated bioactivity-exposure relationship metric and prioritize chemicals for future testing for estrogenic and androgenic activity (EPA, 2015).

IVIVE can also support an informed interpretation of *in vitro* data. Typically, *in vitro* assays target specific mechanistic endpoints (e.g., membrane binding), and therefore are often associated with specific toxicity endpoints. The interpretation of *in vitro* response metrics for a chemical, such as the half-maximal activity concentration (AC50), may vary depending on the chemical, assay mechanism, and decision-making needs. IVIVE facilitates incorporation of this knowledge into decisions by providing *in vivo* context for the results of these assays.

The ability to run IVIVE analyses has generally been limited to users who have coding abilities and an understanding of the models or custom software. This presents a barrier to the user without these skills and knowledge who nevertheless wishes to explore potential applications of IVIVE. This barrier is addressed by tools like ICE, which provides free and open access to a user-friendly interface for generalized PBPK models, along with chemical parameters (experimentally measured and predicted) and curated high-throughput screening (cHTS) *in vitro* assay data. While full code for the ICE PBPK and IVIVE tools is publicly available (https://github.com/NIEHS/ICE_IVIVEpipeline) for those interested in using it, the ICE interface, tools, and data allow users with no coding ability and only a general understanding of PBPK models to estimate EADs based on a variety of user-specified parameters (Bell et al., 2020). Importantly, the cHTS data in ICE are curated to increase data robustness and the assays are linked to mechanistic targets and modes of action to provide additional biological context, both of which facilitate use and interpretation by users new to IVIVE analyses. *Via* the Curve Surfer tool, ICE includes the ability to view and interact with concentration response curves for the cHTS data, allowing users to explore the activity data used for EAD predictions. This feature is key for increasing the transparency of the analyses, as the curves provide experimental context essential for interpreting results, thus encouraging broader use of IVIVE approaches.

## Potential Applications for Regulatory Use

Applications of PBPK modeling and IVIVE can support regulatory decision-making by informing experimental design, incorporating consideration of ADME processes in chemical screening, facilitating the interpretation of bioactivity observed *in vitro*, and enabling margin-of-exposure assessment of *in vitro* assay results.

PBPK modeling estimates plasma and tissue concentrations that result from an external exposure. As these models can simulate *in vivo* ADME across different species and uptake across different exposure routes (Hines et al., 2021), they can inform on relevant doses to use in *in vivo* or *in vitro* experiments. For example, an experiment’s dosing range could be set based on the human plasma concentration estimated from expected exposures and consideration of population variability (Kenyon et al., 2003). This helps ensure that planned experiments cover the concentrations of interest without testing concentrations that far exceed realistic exposures. Accounting for population variability, for example, in the metabolism rates of chemicals, may help identify doses relevant for vulnerable population groups and may allow for more accurate estimation of the safety factors used in traditional risk assessment (Ring et al., 2017; Judson et al., 2018).

PBPK modeling can also inform exposure-based hazard screening. Using QSAR parameters, these models can provide computational predictions of which chemicals may accumulate in the body and which chemicals are likely to be poorly absorbed or cleared quickly. Chemicals that are likely to accumulate may be of greater concern and thus could be candidates for study prioritization. While the applicability domain of QSAR models should be considered during this kind of screening, the results offer insights that would not be possible to obtain by relying solely on experimental data (Kar et al., 2018). Both experimental at QSAR data are available in ICE, U.S. and international regulatory authorities have issued guidance and recommendations for PBPK modeling and development that highlight regulatory applications of PBPK, including extrapolation between species and exposure routes, application of read-across, acute to low-dose extrapolations, and IVIVE (FDA, 1999, 2018; OECD, 2021). It is noteworthy that PBPK models can range from generalized, such as those used in ICE, to highly chemical-specific in nature. Generalized models have the benefit of lower data requirements and can facilitate hazard and risk screening across broad chemical sets, while models constructed, parameterized and calibrated based on individual chemical data may provide more precise results for different questions in regulatory decision making (Zhao et al*.*, 2011). While the simplicity of the generalized models used in ICE can limit predictive precision, it facilitates rapid and broad-reaching application of to inform screening assessments.

In regulatory applications, IVIVE is useful because it provides the *in vivo* (often human) context required to interpret the activity concentration of the biological mechanisms that are targeted by *in vitro* assays. For example, different compounds may have similar bioactivity across a broad array of *in vitro* assays for the same mechanistic target, but IVIVE may result in large differences in human EAD. These differences arise from the incorporation of ADME processes in PBPK modeling. In this way, IVIVE facilitates margin-of-exposure screening by characterizing the difference between a chemical’s expected exposure and its EAD. Chemicals that have small differences between the two values, e.g., less than the standard uncertainty factors used for risk assessment, or even overlapping ranges, become higher priority for further characterization such as chemical-specific models or targeted experimental testing. Current practices in IVIVE analyses often include parameter assumptions such as 100% chemical absorption to provide conservative results. However, additional analysis of uncertainty in PBPK modeling results and subsequent IVIVE can improve the utility of these tools for regulator applications. For example, identifying the maximum and minimum Cmax values, given parameter uncertainty, could allow for better understanding of the confidence regulators can have in results. Additionally, it is worth noting that improved parameter models such as absorption or food binding models have the potential to increase the confidence of IVIVE results for regulatory applications. Future expansions of ICE are underway to incorporate these sources of uncertainty and address other aspects such as variability in chemical metabolism and clearance due to genetic polymorphisms in metabolic enzymes across human populations.

While regulatory guidance documents specific to IVIVE are yet to be developed by most agencies, the application of IVIVE approaches in regulatory contexts has increased dramatically over the last decade. For example, the U.S. Food and Drug Administration promotes the use of NAMs, including PBPK modeling and IVIVE, for filling data gaps to support drug development (Avila et al., 2020). These approaches have also been used for hazard and risk screening of chemicals. In an application relevant to environmental regulation, Wegner et al*.* (2020) used IVIVE to develop an exposure-bioactivity index for endocrine-active chemicals. Recently, Paul-Friedman et al*.* (2020) showed how IVIVE can inform point-of-departure estimation from *in vitro* data and prioritize chemicals for further evaluation using bioactivity:exposure ratios. As agencies move toward reducing dependency on animal testing through the development and use of NAMs, PBPK modeling and IVIVE will be crucial tools to facilitate the interpretation of *in vitro* data.

## Case Study Demonstration

**FIGURE 2:**
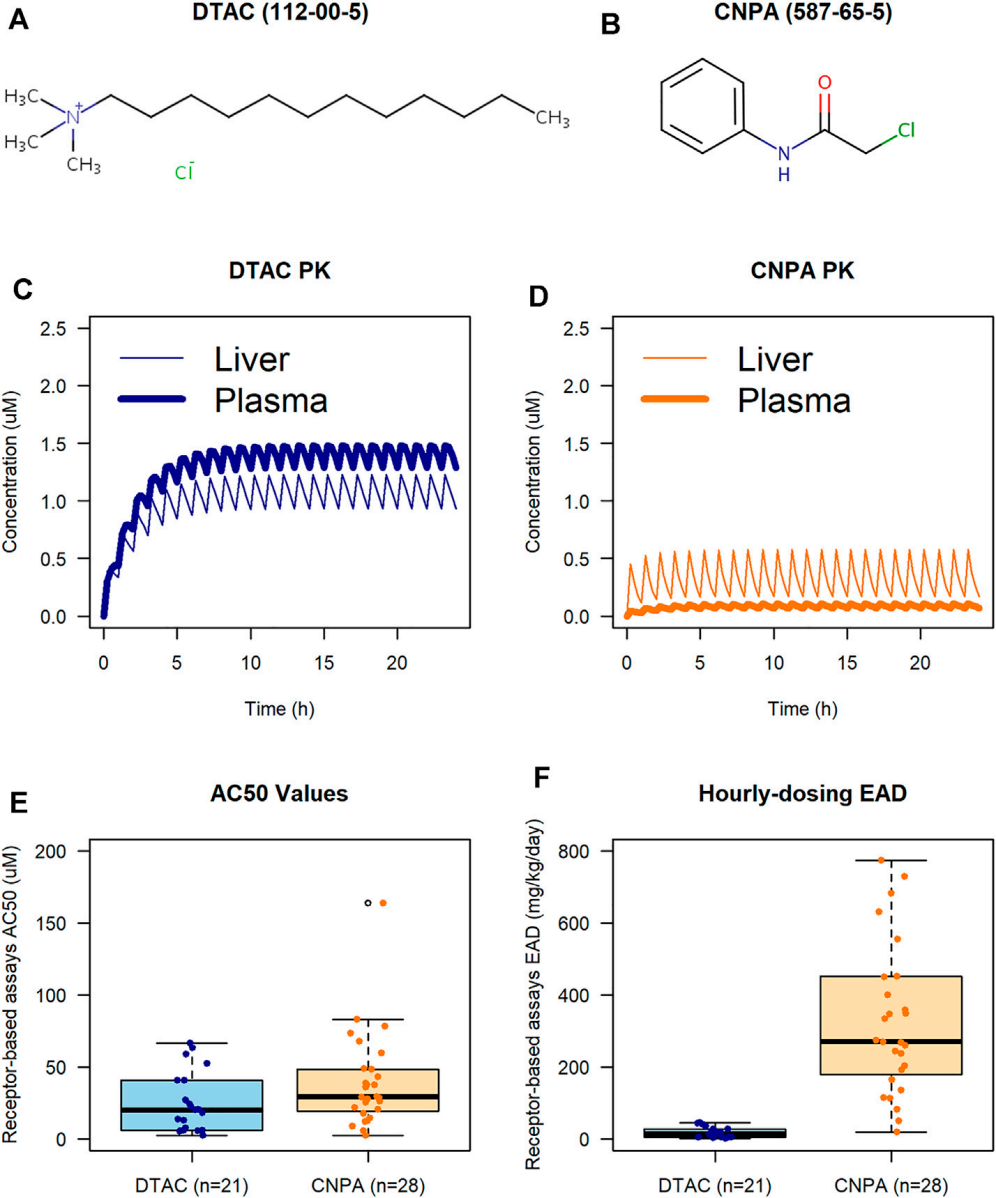
PBPK and IVIVE case study results conducted in ICE. Chemical structures for DTAC and CNPA are from the EPA CompTox Chemical Dashboard [**(A, B)**, respectively; CAS numbers shown in parentheses]. PK profiles show plasma (bold) and liver (plain) concentrations during the simulation for DTAC and CNPA [**(C,D)**, respectively]. AC50 boxplot **(E)** shows similarity between observed bioactivity for receptor-based assays in DTAC and CNPA. Hourly-dosing EAD boxplot **(F)** shows how ADME considerations can result in different EAD predictions for chemicals with similar *in vitro* bioactivity.

To demonstrate the utility of PBPK modeling and IVIVE analyses, we compared two chemicals in a case study: dodecyltrimethylammonium chloride (DTAC; CASRN 112-00-5), a quaternary ammonium compound used in cleaning products, as an emulsifier, and a preservative, and 2-chloro-n-phenylacetamide (CNPA; CASRN 587-65-5), an acetaminophen impurity with laboratory and industrial applications and anti-fungal properties (Silva et al., 2022) that is part of a broader class of chloroacetamides used as herbicides. We selected these chemicals for our case study because they have different chemical structures (Figures 2A,B) and ADME properties but similar *in vitro* bioactivity in receptor-based assays that were identified using the key characteristics of carcinogens (KCC8: Receptor-Mediated Effects) mode of action on ICE. These assays include those involved in receptor activation and/or inactivation or modulation of endogenous ligands including hormones (Smith et al., 2016).

### Physiologically Based Pharmacokinetic Modeling

PBPK modeling of DTAC and CNPA was conducted using workflows accessible *via* ICE to provide an example of how generalized open-source tools can be useful for PK prediction. A detailed description of the model parameters used and the ICE inputs needed to replicate this case study is provided in the Supplementary Material S2. Briefly, we used a PBPK model from the httk R package (Pearce et al., 2017) to simulate an hourly 1 mg/kg body weight oral bolus exposure of each chemical for 24 h in a 70 kg human. We evaluated liver and plasma concentrations to demonstrate how tissue-specific effects resulting from chemical PK properties can influence relevant concentrations.

A key parameter in evaluating chemical metabolism is the maximum concentration of a chemical in plasma (Cmax). Our PBPK modeling results showed that a simulated hourly 1 mg/kg dose of DTAC induced a Cmax that was over 10-fold higher than that induced by an equivalent dose of CNPA (Figures 2C,D; bold lines). This result was largely driven by the accumulation predicted for DTAC after repeat dosing, likely resulting from its relatively low metabolic clearance rates and high tissue partition coefficients (Supplementary Material). When comparing predicted concentrations in the liver, the concentration of DTAC was approximately twice that of CNPA (Figures 2C,D; plain lines). It is notable that the plasma concentration for DTAC was higher than the liver concentration, while the opposite was true for CNPA. Since this case study modeled an oral bolus dose, the chemicals would undergo first-pass metabolism through the liver prior to entering the rest of the body. For DTAC, a relatively low rate of metabolism in the liver (Supplementary Material S2) would mean that much of the chemical would reach the blood stream and be circulated throughout the body. The higher rate of metabolism for CNPA, conversely, would result in less chemical reaching the blood stream for circulation after first-pass metabolism, thus the highest concentrations of CNPA were observed in the liver as this compartment received input directly from the gut prior to metabolic clearance. This prediction demonstrates how the differing ADME properties of these chemicals can influence *in vivo* target tissue concentrations, and the importance of understanding relevant target tissues when interpreting *in vitro* results.

PBPK modeling can also facilitate predictions of tissue-specific *in vivo* chemical concentrations to help identify relevant bioactivity levels through forward dosimetry. Specifically, Cmax estimations predicted to occur from expected exposure conditions can be used to prescribe test ranges. In this case study example, plasma Cmax is predicted to be under 1.5 µM for DTAC and under 0.1 µM for CNPA (under 2 µM; Figures 2C,D).

### *In Vitro* to *In Vivo* Extrapolation

The IVIVE analysis conducted for this case study utilized the plasma Cmax from PBPK modeling results for DTAC and CNPA to estimate EADs based on ICE cHTS assay data. For this example, we used AC50s from assays that measured endpoints affecting receptor-based effects. Receptor-based effects was selected as an endpoint grouping because it was represented by several assays having positive results with similar *in vitro* bioactivity concentrations (AC50) for both chemicals. A full description of the ICE inputs for replicating this case study is provided in the Supplementary Material.

The cHTS data set for “KCC8: Receptor-Mediated Effects” contained 21 active receptor-based assays for DTAC and 28 for CNPA, covering molecular targets relevant to endocrine disruption (e.g., ER and AR), developmental signaling pathways (e.g., RAR) and metabolic pathways involved in immune response and cancer (e.g., VDR, PPARg). For DTAC, the assays in the data set had a median AC50 value of 20.04 µM, while the assays for CNPA yielded a median AC50 value of 29.16 µM. Figure 2E shows that, except for one high outlier for CNPA, all the AC50 values for both chemicals in all assays fell within a similar range. However, the IVIVE analysis (Figure 2F) predicted that, based on the AC50 values for the assay inputs, the median EAD would be 13.53 mg/kg/day for DTAC and 271.26 mg/kg/day for CNPA, a difference of approximately 20-fold.

The EAD predictions generated by IVIVE can be used to derive a margin of exposure by dividing the EAD by an expected daily exposure. For example, the EPA’s exposure forecasting program (ExpoCast; Wambaugh et al., 2013) provides 95th percentile total daily exposure estimates of 0.106 mg/kg and 7.19e-4 mg/kg for DTAC and CNPA, respectively. Thus, the margin of exposure based on the 95th percentile of expected exposure and the median EAD from the input data for this example would be 127.69 for DTAC and 377,274 for CNPA. These unitless metrics provide an estimate of the exposure level expected to result in toxicity relative to anticipated intake, therefore lower values are of greater potential concern. It should be noted that EAD calculations from IVIVE based on Cmax from PBPK modeling are affected by the dosing frequency. Therefore, an accurate representation of relevant exposure in IVIVE analyses is essential to informing decision-making through a margin-of-exposure approach.

It is important to note that the IVIVE analysis simply relates an *in vitro* bioactivity measure to what the estimated *in vivo* plasma concentration would be (or other modeled tissues). It does not indicate whether the concentration is sufficient to cause a sustained *in vivo* response. Evidence in the literature demonstrates that the case study chemicals may have different *in vivo* effects; quaternary ammonium compounds similar to DTAC have been shown to affect neural tube development and fertility in mice at doses of 120 mg/kg/day and (Melin et al., 2014; Hrubec et al., 2017), while there is evidence that a pesticide similar to CNPA can induce enterochromaffin cell tumors in mice at doses above 100 mg/kg/day (Yoshida, 2021). While these experimental results are observed at doses substantially higher than predicted human exposures (Wambaugh et al., 2013), they highlight how these chemicals may affect different *in vivo* outcomes. Additional context about the biological outcome is generally needed to link the mechanistic *in vitro* assays to an adverse outcome, which can potentially be identified through Adverse Outcome Pathways (AOP; Tollefsen et al., 2014; Villeneuve et al., Villeneuve et al.,2014a, Villeneuve et al.,2014b). In the case examples here, bioactive concentrations from a selection of receptor-based assays annotated to “KCC8: Receptor-Mediated Effects” were used in IVIVE (Figure 1). These assays target a diverse group of mainly nuclear receptors, and the overlap between the target chemicals represents a subset of assays that includes androgen, estrogen, and vitamin D receptor targets. While this does not represent a comprehensive picture of the bioactivity of these chemicals, there are clear biological connections between the *in vitro* molecular target activities and the adverse effects observed *in vivo*. A complete table of the assays, AC50 values, and EADs used in the case study is available in Supplementary Appendix A, in addition to the relevant ICE query details and associated metadata.

## Conclusion

The overview and case studies presented here illustrated how user-friendly computational tools such as ICE and OPERA can address common barriers to PBPK modeling and IVIVE and provide a practical understanding of the application of these analyses. Specifically, these open-access tools: 1) expand the range of chemicals available for analysis using QSAR modeling (Mansouri et al., 2018) and curated *in vitro* data (Bell et al., 2020; Abedini et al., 2021), 2) facilitate non-expert application of these techniques through a web interface (https://ice.ntp.niehs.nih.gov/) and walkthrough documentation, and 3) provide transparency with full access to analysis code and modifiable R workbooks. As regulatory applications of NAMs continue to develop, computational tools can facilitate broader understanding and implementation of these approaches.

It is important to note that tools alone are not sufficient for advancing our understanding of potential adverse effects associated with chemical exposure. The case study provided in this work highlights the utility of IVIVE within the Integrated Chemical Environment to better contextualize *in vitro* bioactivity. In this example, the data used were curated by technical experts familiar with both the assay technology and the underlying biology (Bell et al., 2020; Abedini et al., 2021). This approach puts mechanistic *in vitro* data into context both quantitatively (IVIVE) and biochemically (assay metadata) to make the use of NAMs more broadly accessible and interpretable.

## Data Availability

Publicly available datasets were analyzed in this study. This data can be found here: https://ice.ntp.niehs.nih.gov/.

## Abbreviations

AC50, half-maximal activity concentration; ADME, absorption, distribution, metabolism, and excretion; cHTS, curated high-throughput screening; Cmax, maximum plasma concentration; CNPA, 2-chloro-n-phenylacetamide; DTAC, dodecyltrimethylammonium chloride; EAD, equivalent administered dose; HTS, high-throughput screening; httk, high-throughput toxicokinetic; ICE, Integrated Chemical Environment; IVIVE, *in vitro* to *in vivo* extrapolation; NAM, new approach methodology; OPERA, Open (quantitative) structure–activity Relationship App; PBPK, physiologically based pharmacokinetic; PK, pharmacokinetics; QSAR, quantitative structure-activity relationship; EPA, U.S. Environmental Protection Agency; NICEATM, National toxicology program’s Interagency Center for the Evaluation of Alternative Toxicological Methods.
