# Supplementary material for: Application of an Accessible Interface for Pharmacokinetic Modeling and In Vitro to In Vivo Extrapolation
Source: Front Pharmacol. 2022 Apr 13;13:864742. doi: 10.3389/fphar.2022.864742 (PMC9043603; doi:10.3389/fphar.2022.864742)
Supplement: Supplementary file 2 [file DataSheet1.docx]

Supplementary material

This document contains supplementary material for the manuscript titled “Application of an Accessible Interface for Pharmacokinetic Modeling and In Vitro to In Vivo Extrapolation”

# S.1 QSAR parameter estimation

The predicted parameters used for the case studies described in this work were generated by the Open (quantitative) structure–activity Relationship App (OPERA). OPERA is an open-source open-data suite of quantitative structure–activity relationship (QSAR) models developed and maintained under an ongoing collaboration between NICEATM and the EPA’s Center for Computational Toxicology and Exposure (Mansouri *et al.*, 2018). OPERA provides predictions for a number of physicochemical and ADME properties as well as environmental fate and toxicity endpoints. All models in OPERA were developed using highly curated experimental datasets and QSAR-ready chemical structures generated by an automated standardization workflow (Mansouri *et al.*, 2016). In addition to property predictions, OPERA provides applicability domain assessment and accuracy estimates following the five OECD principles for QSAR validation (Tichý and Rucki, 2009; Mansouri *et al.*, 2018). The current OPERA version (v2.7) can be downloaded as a command-line or a user-friendly graphical interface from the official NIEHS GitHub repository (<https://github.com/NIEHS/OPERA>).

OPERA generates predictions of ADME-related properties used for PBPK and IVIVE modeling, including plasma protein fraction unbound (F_U_), intrinsic clearance (CL_int_), octanol-water partition coefficient (LogKow), dissociation coefficient (LogD), Henry’s Law constant (HL) and the logarithmic acid dissociation constant (pKa).

## S.1.1 Plasma fraction unbound and intrinsic clearance

Both F_U_ and CL_int_ models in OPERA were built on experimental data included in the latest version of the EPA’s httk package (Pearce *et al.*, 2017; Wambaugh *et al.*, 2019) combined with other public sources (Zhu *et al.*, 2013; Ingle *et al.*, 2016; Da-silva *et al.*, 2018; OECD, 2020). The data used to build the models were curated in multiple steps. First, the sources were checked to correct or remove erroneous and extreme values. Then, representative values were calculated for the chemicals, with the median of the verified values becoming the assigned value for chemicals with multiple entries. Finally, the initial outlier removal step was repeated to remove outliers among the representative values. After several rounds of automated and manual curation steps to reduce errors, variability, and outliers, the F_U_ and CL_int_ data sets consisted of 1056 and 1873 chemicals, respectively.

These data sets were split into training (75%) and test (25%) sets keeping a similar distribution of the experimental values between the subsets. Different machine learning algorithms were tested, and it was found that the weighted nearest neighbors (kNN) method provided the best predictive performance while keeping the algorithm simple and interpretable as recommended by the OECD principles. The F_U_ model was based on a simple regression model. However, modeling the CL_int_ data required a hybrid two-stage approach. In this approach, a classification model is first used to separate the cleared from non-cleared chemicals. Then, a regression model is applied to predict the CL_int_ value for the cleared chemicals. The statistics for the models are reported in Tables S1 and S2.

Table S1. Statistics for the F_U_ model in five-fold cross-validation and external test set.

|  | **Cross-validation** | **Test** |
| --- | --- | --- |
| R^2^ | 0.63 | 0.65 |
| RMSE | 0.20 | 0.19 |

Table S2. Statistics for the CL_int_ model in five-fold cross-validation and external test set.

|  | **Cross-validation** | **Test** |
| --- | --- | --- |
| BA | 0.70 | 0.57 |
| R^2^ | 0.40 | 0.39 |
| RMSE | 0.73 | 0.79 |

## S.1.2 Tissue partition coefficients

The tissue partition calculations required by the PBPK/IVIVE pipeline are based on the predictions generated by OPERA models for the parameters LogKow, LogD, pKa, and HL.

The LogKow and HL models were two of the first endpoints for which models were available in the OPERA suite (Mansouri et al 2018). These models were recently updated with additional curated data for some groups of chemicals based on the relevance to environmental studies reaching a total number of 13,936 and 697 unique QSAR-ready structures for LogKow and HL, respectively. In predicting the test set properties, the LogKow model had an R2 of 0.86 and an RMSE of 0.78, while the HL model had an R2 of 0.85 and an RMSE of 1.82.

The OPERA pKa model was developed based on a data set of 7,912 chemicals as described by Mansouri *et al.* (2019) and predicts a chemical's most acidic and most basic pKa values. The acidic and basic pKa models reached R^2^ values of 0.72 and 0.78 and RMSE of 1.80 and 1.53, respectively.

LogD is the distribution coefficient that takes into account pH-dependence and is used to estimate the different relative concentrations of the ionized and non-ionized forms of a chemical at a given pH. OPERA uses both pKa and logKow predictions to provide logD estimates for ionizable chemicals at pH 5.5 and pH 7.4 using the following formula:

$${logD}_{(pH)}=logP-\log(1+{10}^{\left( pH-pKa \right)})$$

These parameters were then used in conjunction with the Schmitt (2008) implementation in the httk package (Pearce *et al.*, 2017) to calculate tissue partition coefficients based on the httk tissue composition values. The Yun and Edginton (2013) formulation was used to predict membrane affinity during partition coefficient calculations as necessary.

# S.2 Case study inputs

We used a multi-compartment human PBPK model (solve_pbtk) from the httk R package (Pearce *et al.*, 2017), which includes compartments for arteries, veins, and selected other tissues (GI tract, liver, lungs, and kidneys). Remaining tissues were combined into one “rest-of-body” compartment. We modeled an oral bolus exposure route (gut absorption) and simulated a 70kg human dosed with 1 mg/kg body weight of each chemical every hour for 24 hours.

## S.2.1 PBPK modeling

PBPK modeling and IVIVE was conducted for a case study of dodecyltrimethylammonium chloride (DTAC; CASRN 112-00-5) and 2-chloro-n-phenylacetamide (CNPA; CASRN 587-65-5). The ICE user PBPK tool interface input screen included the following inputs:

- Species = human
- ADME source = default
- Output Units = uM
- Model = Solve_pbtk
- Exposure route = oral
- Simulation length = 1 day

Additionally, inputs for dosing included:

- Exposure interval=1 hour (corresponding to 24 doses per day)
- Exposure dose=0.0416667 (1/24; total dose of 1 mg/kg/day)

Chemical-specific ADME parameters (including Clint, Fub, and tissue partition coefficients) were obtained from OPERA predictions. The parameters used for this case study, which are drawn from ICE data based on user inputs, can be seen in Table S3. This case study focused on both plasma and liver concentrations; plasma concentrations were used for IVIVE analysis, while liver concentrations were reported to demonstrate the tissue-dependent effects of ADME properties.

Table S3: Example input parameters used for the case study in this work.

| **Parameters** | **Parameter Description** | **DTAC** | **PNAC** |
| --- | --- | --- | --- |
| BW | body weight (kg) | 70 | 70 |
| Clint | in vitro intrinsic clearance (uL/min/10^6 cells) | 16.2 | 38.9 |
| Fgutabs | fraction of the oral dose absorbed | 1 | 1 |
| Fhep.assay.correction | fraction of chemical unbound in hepatocyte assay using the method of Kilford et al. (2008) | 0.9397 | 0.9321 |
| Funbound.plasma | fraction of a chemical in plasma that is not bound to plasma protein | 1 | 0.889 |
| hematocrit | percent volume of red blood cells in the blood | 0.44 | 0.44 |
| Kgut2pu | ratio of concentration of chemical in gut tissue to unbound concentration in plasma | 4.92 | 5.247 |
| kgutabs | rate per hour that chemical enters the gut from gut lumen | 2.18 | 2.18 |
| Kkidney2pu | ratio of concentration of chemical in kidney tissue to unbound concentration in plasma | 15.72 | 9.696 |
| Kliver2pu | ratio of concentration of chemical in liver tissue to unbound concentration in plasma | 21.46 | 11.31 |
| Klung2pu | ratio of concentration of chemical in lung tissue to unbound concentration in plasma | 4.748 | 2.31 |
| Krbc2pu | ratio of concentration of chemical in red blood cells to unbound concentration in plasma | 0.7711 | 1.43 |
| Krest2pu | ratio of concentration of chemical in rest of body tissue to unbound concentration in plasma | 4.702 | 3.454 |

The ICE PBPK tool returns results in the form of downloadable files, which were used to create Figure 2 in the manuscript associated with this supplemental file, as well as in the form of customizable graphs. Figure S 1 shows the PBPK tool graphical output for CNPA.


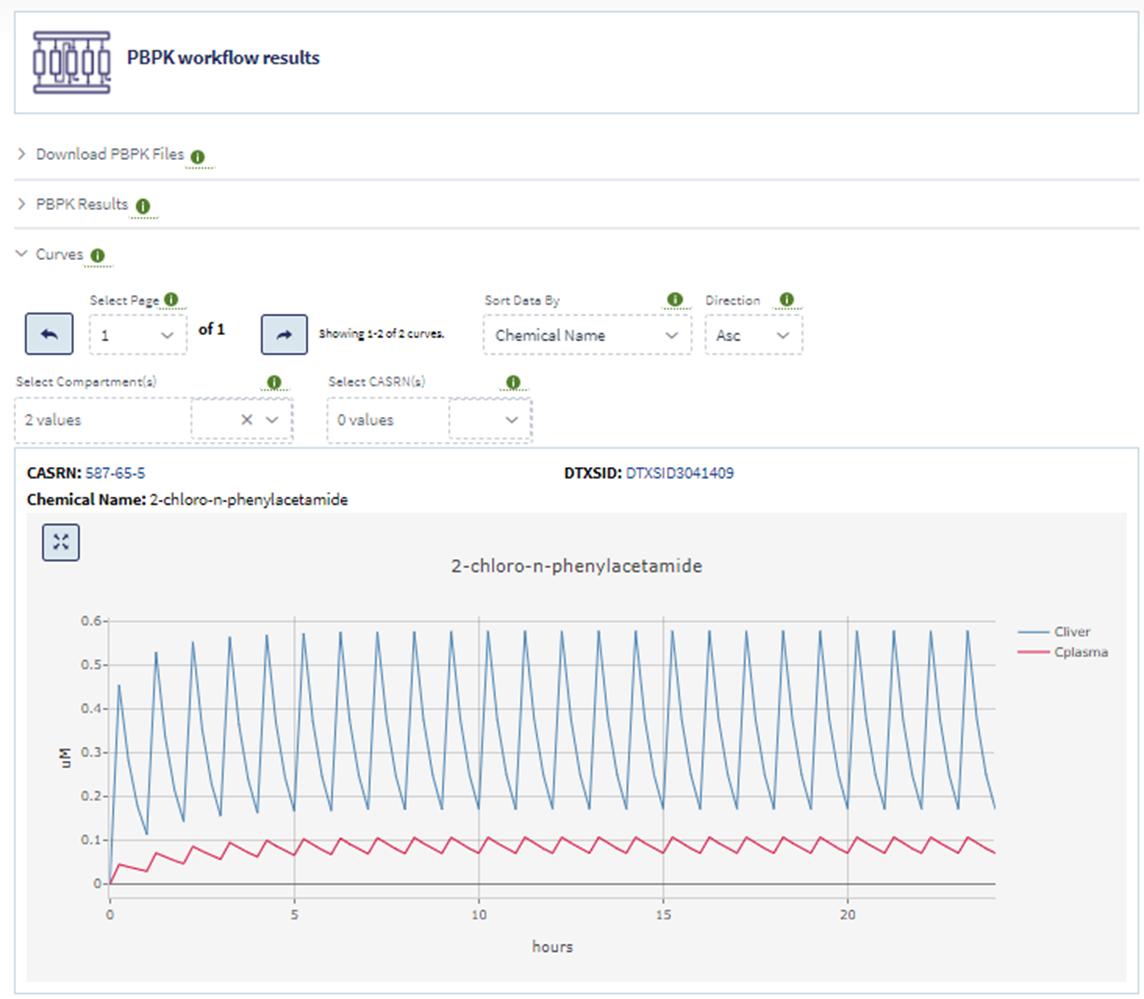


Figure S 1: Screen-capture of PBPK tool output for CNPA (CASRN 587-65-5) taken on March 10, 2022. Plasma concentrations are shown in red, while liver concentrations are shown in blue. Plots in ICE are fully zoomable and interactive, and can be customized using dropdown menus.

## S.2.2 IVIVE

For the ICE IVIVE analysis we used the following inputs:

- In vitro endpoint = AC50
- Species = human
- ADME source = default
- Model = Solve_pbtk
- Exposure route = oral
- Exposure interval =1 hour
- Simulation length = 1 day

Additional dosing inputs are not necessary for the ICE IVIVE tool, as a dose of 1 mg/kg/day is assumed. The category "KCC8: Receptor Mediated Effects", which can be found under the Cancer mode of action tab, was used for in vitro assay input. The EAD output provided by ICE is in units of mg/kg/dose. Therefore, we multiplied all EAD values by 24 doses/day to obtain the predicted EAD in units of mg/kg/day.

As with the PBPK tool, the ICE IVIVE tool provides output in the form of both downloadable tables and interactive plots. An example output plot showing EAD values (mg/kg/dose) can be seen in Figure S 2.


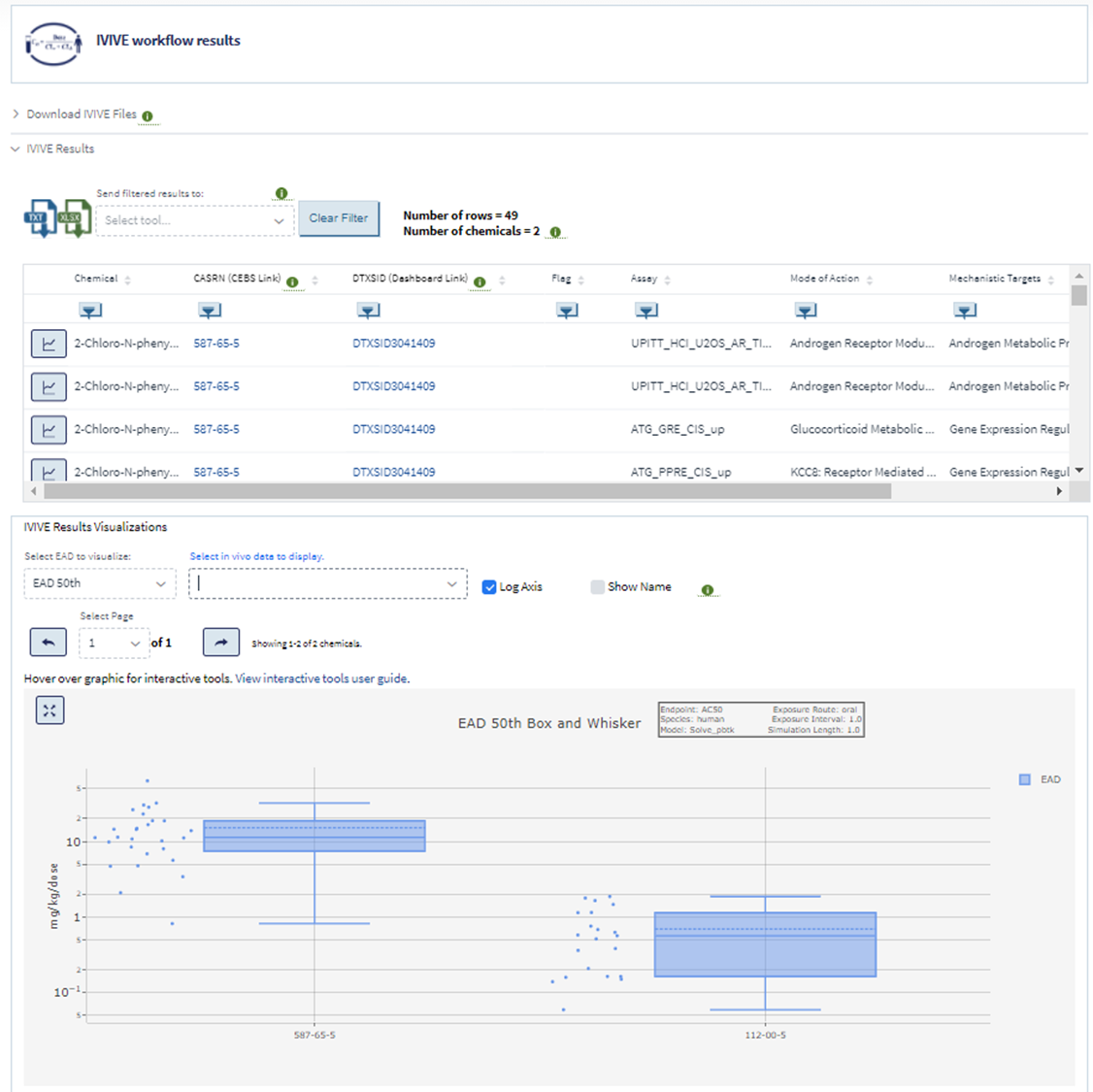


Figure S 2 Screen-capture of IVIVE tool output for CNPA (CASRN 587-65-5) and DTAC (CASRN 112-00-5) taken on March 10, 2022. Note the log10 y-axis scale, which differs from that of Figure 2 in the manuscript associated with this supplement.

# S.3 Appendix A

The complete table of in vitro assays, bioactivity data (AC50), and EAD values for both DTAC and CNPA are available in the attached appendix as a Microsoft Excel workbook. ICE users have the option to download similar tables after every ICE query. There are three tabs in the workbook: a MetaData tab describing the contents of each page, an IVIVE Results tab providing the analysis results, and an ICE Query tab documenting the inputs to the analysis. The columns present in the IVIVE results tab are as follows:

- Chemical: chemical name
- CASRN: CAS registration number
- DTSXID: Chemical identification number
- Assay: Name of assay
- MOA: Modes of action linked to assay
- Mechanisms: Mechanisms linked to assay
- AC50 (uM): Bioactivity value (AC50, uM)
- EAD 50^th^ Percentile (mg/kg/dose): Effective administered dose (mg/kg/dose)
- EAD 50^th^ Percentile (mg/kg/day): Effective administered dose scale to daily intake as presented in the associated manuscript (mg/kg/day)

# References

Da-silva, F. *et al.* (2018) ‘Improving Prediction of Metabolic Clearance Using Quantitative Extrapolation of Results Obtained From Human Hepatic Micropatterned Cocultures Model and by Considering the Impact of Albumin Binding’, *Journal of Pharmaceutical Sciences*, 107(7), pp. 1957–1972. doi:10.1016/j.xphs.2018.03.001.

Ingle, B.L. *et al.* (2016) ‘Informing the human plasma protein binding of environmental chemicals by machine learning in the pharmaceutical space: applicability domain and limits of predictability’, *Journal of chemical information and modeling*, 56(11), pp. 2243–2252.

Mansouri, K. *et al.* (2016) ‘An automated curation procedure for addressing chemical errors and inconsistencies in public datasets used in QSAR modelling’, *SAR and QSAR in Environmental Research*, 27(11), pp. 911–937. doi:10.1080/1062936X.2016.1253611.

Mansouri, K. *et al.* (2018) ‘OPERA models for predicting physicochemical properties and environmental fate endpoints’, *Journal of Cheminformatics*, 10(1), p. 10. doi:10.1186/s13321-018-0263-1.

Mansouri, K. *et al.* (2019) ‘Open-source QSAR models for pKa prediction using multiple machine learning approaches’, *Journal of Cheminformatics*, 11(1), p. 60. doi:10.1186/s13321-019-0384-1.

OECD (2020) ‘Case study on the use of Integrated approaches to testing and assessment for read-across based filling of developmental toxicity data gap for methyl hexanoic acid’. OECD. Available at: https://www.oecd.org/officialdocuments/publicdisplaydocumentpdf/?cote=ENV/JM/MONO(2020)21&docLanguage=en.

Pearce, R. *et al.* (2017) ‘httk: R Package for High-Throughput Toxicokinetics’, *Journal of Statistical Software*, 79(4). doi:10.18637/jss.v079.i04.

Schmitt, W. (2008) ‘General approach for the calculation of tissue to plasma partition coefficients’, *Toxicology in Vitro*, 22(2), pp. 457–467. doi:10.1016/j.tiv.2007.09.010.

Tichý, M. and Rucki, M. (2009) ‘Validation of QSAR models for legislative purposes’, *Interdisciplinary Toxicology*, 2(3), pp. 184–186. doi:10.2478/v10102-009-0014-2.

Wambaugh, J.F. *et al.* (2019) ‘Assessing Toxicokinetic Uncertainty and Variability in Risk Prioritization’, *Toxicological Sciences*, 172(2), pp. 235–251. doi:10.1093/toxsci/kfz205.

Yun, Y.E. and Edginton, A.N. (2013) ‘Correlation-based prediction of tissue-to-plasma partition coefficients using readily available input parameters’, *Xenobiotica*, 43(10), pp. 839–852.

Zhu, X. *et al.* (2013) ‘The Use of Pseudo-equilibrium Constant Affords Improved QSAR Models of Human Plasma Protein Binding’, *Pharmaceutical research*, 30(7), pp. 1790–1798. doi:10.1007/s11095-013-1023-6.
